# Proceedings of the Mississippi Valley Dental Association

**Published:** 1892-05

**Authors:** 


### PROCEEDINGS.

### Proceedings of the Mississippi Valley Dental Association.

The Forty-eighth Annual Meeting of the Mississippi Valley Dental Association was held in the Lincoln Club Hall, Eighth and Race streets, Cincinnati, O., on Wednesday, March 9, 1892.

The Society was called to order by the President, L. E. Custer, D. D. S., of Dayton, O., at 10:30 A. M.

The minutes of the last meeting were read by the Recording Secretary, H. T. Smith, and approved.

The name of Dr. A. A. Kumler, of Cincinnati, was presented by Dr. C. M. Wright, for membership, the rules were suspended, the Secretary cast the ballot and Dr. Kumler was elected.

The report of the Treasurer was then read, and a recess granted members for the payment of dues.

Owing to the small number in attendance at this hour, a motion was made to postpone the President's address until the afternoon session, which was afterwards withdrawn. Dr. Jessie Dillon, Second Vice-President, taking the chair during the President's address; which was followed by a discussion, on the feasibility of the co-operation of the Fiftieth Anniversary of this Society with the World's Columbian Dental Congress, which is to be held next year in Chicago. A Nominating Committee was then appointed to consist of Drs. Fletcher, Wright and Taft, who were to select suitable persons to look up the literature pertaining to the history of this Society.

Dr. Hunter moved that the morning session be from 9 to 12, the afternoon from 2 to 5, and no evening session, except on Thursday, which evening had been set apart for Dr. Fletcher's paper illustrated by lantern. Carried.

There being no further business at this hour—adjourned till 2 P. M.

### WEDNESDAY AFTERNOON SESSION.

Meeting called to order at 2 o'clock by President Custer.

Minutes of the morning session read and adopted.

Owing to the unavoidable absence of Drs. Callahan and Sage, whose names appeared first on the programme, it was moved and seconded that Dr. C. M. Wright, of Cincinnati, read his paper "On Edge". Dr. Wright rose with the remark, "I wish to stand up as a man ready prepared, even if it is not my turn." This paper was followed by a very lengthy and amusing discussion as to the different methods of rest and recuperation from nervousness, caused by continual standing at the chair, in which Drs. Hunter, Smith, Morrison, Fletcher, R. E. Taylor and others took part.

Dr. W. B. Ames, of Chicago, Ills., then read his paper entitled, "A New Oxyphosphate for Crown Setting", in which were brought out many points in favor of this new cement for filling cavities, etc., its desirable working qualities, its hardness and stability after crystallization, also the length of time it remains in the plastic condition, giving the operator plenty of time. This paper was discussed by Drs. Cassidy, Heise, Morrison, Smith, Wright and Ames.

Drs. Leslie, Cassidy and Fletcher were then appointed a Committee on Necrology, to draw up resolutions on the death of J. G. Cameron, D. D. S., of Garfield Place, Cincinnati.

Dr. C. M. Wright then extended a cordial invitation to all present to attend the Commencement Exercises of the Ohio Dental College at the Odeon that evening.

Adjourned to 9 o'clock Thursday.

THURSDAY MORNING, MARCH 10TH, 9 A. M.

Meeting called to order by the President.

Minutes of previous session read and approved.

Dr. F. W. Sage, of Cincinnati, not being present at this time, it was moved and seconded that his paper be read by the Secretary.

Dr. Sage's long and interesting paper on "Discretion in the Performance of Dental Operations" was then read; the discussion of which was opened by Dr. H. A. Smith, followed by some remarks by Dr. Sage.

Dr. G. S. Junkerman, of Cincinnati, read a paper on "Surgery of the Antrum," in which was clearly brought out the advisability of extracting the fang of the tooth, should it by accident break off and be forced into the Antrum. It was ably discussed by Drs. Knight, Wright, Jay, Junkerman, Smith and Fletcher.

Dr. H. A. Smith, of Cincinnati, then read a paper on "Injury to the Superior Incisor Teeth; Supposed Agency of Phagocytes in Effecting a Cure." The discussion opened by Dr. Wright, followed by Drs. Cassidy, Heise, Fletcher, Smith and Hunter.

A meeting of the stockholders of the Ohio College at 1:30 P. M., was then announced.

Adjourned to the afternoon.

THURSDAY AFTERNOON, MARCH 11TH, 2 P. M.

After the meeting of stockholders at 1:30, the regular session was called to order by the President at 2 o'clock.

Minutes of the previous session read and approved.

A voluntary paper by Wm. Knight, M. D., of Cincinnati, on "Permanent Closure of the Jaws." An exhibition of the patient, (who had been suffering for fifteen years, and during that time had existed on liquids taken through a vacuum made by knocking out two of the teeth,) upon whom this delicate and successful operation had been performed, added much to the interest of the meeting.

Discussion by Drs. Harlan, H. T. Smith, Knight and Leslie.

Dr. Grant Mollyneaux, of Cincinnati, also read a most interesting paper on "Mechanical Treatment of Cleft Palate," illustrated by patients, models and charts. Dr. Mollyneaux demonstrating the hygienic properties of the Hard Rubber for these Appliances. Discussion by Drs. Ames, Doyle, Smith and Morrison.

The Committee on Membership then proposed the names of Mrs. Dr. Montz, of Warsaw, Ky., and Dr. B. F. Johnson, of Camden, O. Rules suspended and they were elected by ballot.

THURSDAY EVENING SESSION.

Called to order by President Custer at 8 P. M.

Notice that the meeting on Friday morning would be at the Ohio Dental College at 9 o'clock.

Dr. M. H. Fletcher's paper on "The Skin and its Appendages, with Special Reference to the Development of the Teeth," illustrated by lantern, was both interesting and instructive.

A vote of thanks was tendered Dr. Fletcher for his valuable paper.

Adjourned to 9 A. M. Friday.

### FRIDAY MORNING SESSION.

Meeting called to order by President Custer.

Minutes of the afternoon session read and approved.

The first paper was by Dr. A. O. Rawls, of Lexington, Ky., on "Decolorization of the Teeth." Discussion by Drs. Taft, Baxter, Cassidy and Heise."

Dr. O. N. Heise, of Cincinnati, read a paper on "Pental, the New Anæsthetic." in which was brought forth many strong points in favor of this new preparation for use in all surgical operations. Discussion opened by J. S. Cassidy, of Covington, followed by nearly all present.

Resolutions were then read on the death of Dr. Cameron, approved, and placed on the minutes, a copy ordered to be sent to the bereaved family, also published in the daily papers; after which fifteen minutes were spent in Memorial Services. All spoke of his noble character, expressed their deep sorrow at his death and realized they had lost a true friend.

The Society next proceeded to the election of officers for the ensuing year. President, O. N. Heise, Cincinnati; First Vice-President, W. B. Ames, of Chicago; Second Vice-President, A. Rose, of Cincinnati; Recording Secretary, H. T. Smith, of Cincinnati; Corresponding Secretary, H. C. Matlack, of Cincinnati; Treasurer, F. A. Hunter, of Cincinnati.

The President-elect was conducted to the Chair and introduced to the Society by Dr. Hunter; after which the meeting adjourned to 2 o'clock.

### AFTERNOON SESSION.

Interesting Clinics were held in the afternoon. Demonstration of the use of the Bonwill Mechanical Mallet, with Electric Motive Power; and other things.

Adjourned to meet next year in the Ohio College of Dental Surgery.

